# 0104. Modulatory effects of heat shock with or without glutamine compared to LPS on peripheral blood mononuclear cells heat-shock-protein 90α expression in severe sepsis and trauma

**DOI:** 10.1186/2197-425X-2-S1-P14

**Published:** 2014-09-26

**Authors:** E Briassouli, M Tzanoudaki, G Daikos, K Vardas, M Kanariou, C Routsi, S Nanas, G Briassoulis

**Affiliations:** 1st Department of Propaedeutic InternalMedicine, University of Athens, Athens, Greece; Department of Immunology - Histocompatibility, Specialized Center & Referral Center for Primary Immunodeficiencies - Paediatric Immunology, “Aghia Sophia” Children's Hospital, Athens, Greece; First Critical Care Department, University of Athens, Evangelismos Hospital, Athens, Greece; PICU, University of Crete, University Hospital, Heraklion, Greece

## Introduction

Inflammatory stimuli cause posttranslational modifications of inducible 90α-kDa-heat-shock-protein (HSP90α) that are Hsp90-inhibitor sensitive and may be important to the pro-inflammatory actions of Hsp90α.

## Objectives

We investigated the heat-shock (HS) and lipopolysaccharide (LPS)-stress response effect on HSP90α in cultured peripheral blood mononuclear cells (PBMCs) from patients with severe sepsis (SS) or trauma-related systemic inflammatory response syndrome (SIRS) compared to healthy-controls (H) and any possible modulating Glutamine (Gln)-effect.

## Methods

PBMCs of 16/H, 11/SS, and 7/SIRS were incubated with 1µg/ml LPS or 43^o^HS vs.no stimulation for 4h. In each group 3 experiments involved L-Ala-Gln10mM incubation 1h before (Gln-b) or after (Gln-a) induction, or no glutmanine (1088 measurements). Intracellular Mean Fluorescence Intensity (MFI) levels of monocytes (mHSP90α) or lymphocytes (lHSP90α) determined using Flow Cytometry.

## Results

Baseline mHSP90α was higher in SIRS (187±30 vs. 112±10, p< 0.01) and lHSP90α in SS (91±19 vs. 47±3, p< 0.001) compared to H. LPS induced H-mHSP90α (141±12 vs. 112±10, p< 0.001) and HS H-lHSP90α (66±7 vs. 47±3, p< 0.0001). Neither LPS nor HS exhibit any significant effect in SIRS- or SS-mHSP90α or lHSP90α. Glutamine given before LPS suppressed SS-lHSP90α (Gln-b 61±5 vs. 91±19, p< 0.004). Similarly, when glutamine was given before or after HS suppressed SS-lHSP90α (Gln-a 73±5 vs. 91±19, p< 0.001; Gln-b 78±4 vs. 91±19, p< 0.05), respectively.

## Conclusions

PBMCs express higher baseline mHSP90α in SIRS and lHSP90α in SS, not further induced by LPS or HS, contrasting their induction effects in H. Gln pre-treatment may attenuate the LPS or HS-induced lHSP90α in SS.

